# A new mathematical modeling approach for thermal exploration efficiency under different geothermal well layout conditions

**DOI:** 10.1038/s41598-021-02286-z

**Published:** 2021-11-25

**Authors:** Junyi Gao, Qipeng Shi

**Affiliations:** 1 School of Architecture and Civil Engineering, Yan’an University, Yan’an, 716000 China; 2 Shandong Provincial Lunan Geology and Exploration Institute, Jining, 272100 China; 3 Shandong Geothermal Clean Energy Exploration and Development Engineering Research Center, Jining, 272100 China

**Keywords:** Hydrology, Environmental impact

## Abstract

The water temperature at the outlet of the production well is an important index for evaluating efficient geothermal exploration. The arrangement mode of injection wells and production wells directly affects the temperature distribution of the production wells. However, there is little information about the effect of different injection and production wells on the temperature field of production wells and rock mass, so it is critical to solve this problem. To study the influence mechanism of geothermal well arrangement mode on thermal exploration efficiency, the conceptual model of four geothermal wells is constructed by using discrete element software, and the influence law of different arrangement modes of four geothermal wells on rock mass temperature distribution is calculated and analyzed. The results indicated that the maximum water temperature at the outlet of the production well was 84.0 °C due to the thermal superposition effect of the rock mass between the adjacent injection wells and between the adjacent production wells. Inversely, the minimum water temperature at the outlet of the production well was 50.4 °C, which was determined by the convection heat transfer between the water flow and the rock between the interval injection wells and the interval production wells. When the position of the model injection well and production well was adjusted, the isothermal number line of rock mass was almost the same in value, but the direction of water flow and heat transfer was opposite. The study presented a novel mathematical modeling approach for calculating thermal exploration efficiency under various geothermal well layout conditions.

## Introduction

In the process of geothermal exploration, if the limited groundwater resources around the geothermal well cannot replenish pumping capacity through runoff, it is then necessary to consider the injection well. This is replenish production well-pumping capacity in time to achieve the dynamic balance between pumping capacity and injection capacity, allowing for long-term geothermal exploration. Underground hot water can be used for heating and generating power after being pumped to the ground. The geothermal water extraction system is affected not only by the groundwater flow field and temperature field but also by the layout of geothermal wells and many other factors. Under the combined effect of these factors, how injection wells and production wells are scientifically and reasonably arranged has a significant impact on the temperature field of the rock mass near the production wells and well groups. Therefore it is of great engineering significance to study the wellbore temperature field in the exploration and development of geothermal resources^1–3^.

At present, research on geothermal well temperatures primarily focuses on numerical simulation analysis. Many scholars have researched the influencing factors of fluid, rock temperature field and wellbore temperature^4^, the influence of groundwater flow velocity in sandy aquifer on the thermal performance of borehole heat exchanger^5^, three-dimensional thermoporoelastic modeling and analysis of flow, heat transport and deformation in fractured rock with applications to a lab-scale geothermal system^6^ and numerical simulation analysis on the influence of different factors on the thermal distribution around wellbore^7^. Groundwater flow estimation for temperature monitoring in borehole heat exchangers during thermal response tests^8^, heat extraction analysis of a novel multilateral-well coaxial closed-loop geothermal system^9^ and research on the influence of borehole heat-water exchanger characteristics on the performance of vertical closed-loop ground heat pump systems were carried out^10^. Gao^11,12^ studied the influence mechanism of geothermal well spacing, geothermal temperature and production well depth on the water flow and heat transfer temperature of rock masses, but the literature did not consider the influence of the interaction of injection wells and production wells on the temperature field of production wells and rock masses. Research on outlet temperature and temperature field of geothermal well^13–16^, sensitivity analysis of influencing factors for heat loss of geothermal wells^17^ and wellbore temperature loss model and application for heating geothermal mining^18^. However, the research contents of these scholars did not involve the comparative study of the water temperature and temperature field at the outlet of geothermal wells under different conditions of the water inlet and water outlet. Scholars have carried out researches on the influence of pumping and irrigation well layout on the groundwater flow field and temperature field^19^, the influence of pumping and irrigation well distribution mode, and pumping and irrigation well water quantity on heat transfer characteristics of underground heat exchanger well^20,21^, and the application of numerical simulation of water and heat transport to optimize pumping and irrigation well the layout of groundwater source heat pump system^22^, numerical simulation of water-heat coupling of single well ground water source heat pump in T2Well^23^ and optimization of reasonable well spacing and layout of shallow source heat pump simulated by sand tank-taking Jiuxi in Fenglin as an example^24^. Sustainable electricity generation from an enhanced geothermal system were carried out considering reservoir heterogeneity and water losses with a discrete fracture model^25^ and enhanced geothermal systems (EGS): hydraulic fracturing in a thermoporoelastic framework^26^ and modified zipper fracturing in an enhanced geothermal system reservoir and heat extraction optimization via orthogonal design^27^. Again, Xu et al.^28^ Studied on optimal arrangement of pumping and irrigation systems for a groundwater heat pump. Deng et al.^29^ conducted a simulation study on the optimization of middle-deep geothermal recharge wells based on optimal recharge efficiency. Olabi et al.^30^ thought that geothermal-based hybrid energy systems are an energy method towards eco-friendliness. Rezaei et al.^31^ researched an enviro-economic optimization of a hybrid energy system from biomass and geothermal resources for low-enthalpy areas. The system off-design evaluation of geothermal-solar hybrid power and operational strategies for its heat pump system was studied^32,33^. Tian et al.^34^ studied Carbon–neutral hybrid energy systems with deep water source cooling, biomass heating, and geothermal heat and power. Chen et al.^35^ carried out Thermodynamic performance analysis and multi-criteria optimization of a hybrid combined heat and power system coupled with geothermal energy. In summary, although some achievements have been made in the study of geothermal well temperature, there are few reports on the complex model of thermal recovery efficiency optimization under different geothermal well layout conditions. The actual geothermal mining process is closely related to the scientific and reasonable layout of geothermal wells. The influence of different geothermal well layout conditions on the temperature field of production wells and rock masses is directly related to the safety and efficiency of geothermal mining. Given this, it is necessary to research the optimization of thermal mining efficiency under different geothermal well layout conditions.

In this paper, first, the fractured rock mass models of four injection wells and production wells are constructed by 3DEC discrete element software. The effect of different water inlets and outlets on the temperature field of the production well and rock mass, as well as the water temperature of the production well outlet, is then calculated under various geothermal well layout conditions. Finally, through comparative analysis, the law of the influence of different geothermal well layouts on the rock mass water flow and heat transfer temperature is revealed.

## Conceptual model of geothermal exploitation

**Figure 1:**
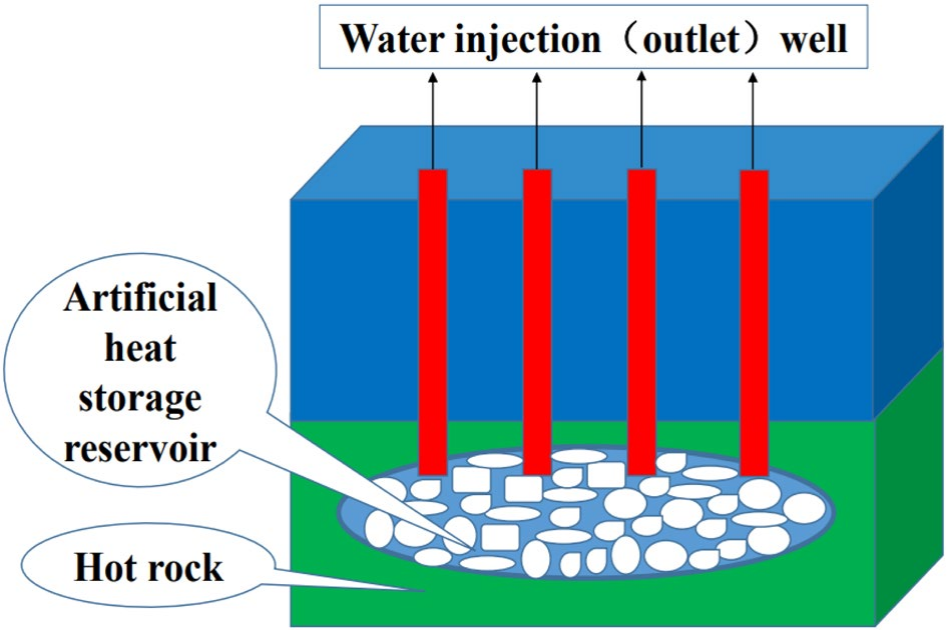
Schematic diagram of geothermal resource exploitation.

Figure 1 shows a schematic diagram of geothermal resource exploitation. Four water injection wells and water output wells were drilled from the ground by using mechanical drills. The hot rock area at the bottom of the water injection wells and water output wells was mechanically fractured to form a microjoint system to open its fractured channel. The ground injected low-temperature water into the water injection well, and the water flowed into the well's bottom. Hot water is stored in the artificial heat reservoir area through convection and heat transfer with high-temperature hot rock, and high-temperature water is pumped out to the ground through the well for comprehensive utilization, such as power generation and heat. In this paper, only four injection wells and production wells are considered, and engineering fracture systems are ignored.

## Basic assumptions of the model

The variables involved in heat conduction in 3DEC are temperature and the three components of the heat flux. The energy balance equation and Fourier law of heat conduction are related to these variables. The differential equation of heat conduction is obtained by combining the Fourier law with the energy balance equation. The differential equation can be solved under specific boundary and initial conditions based on specific geometry and properties. The following dimensionless numbers are used to characterize transient heat conduction.

Characteristic length:

1 $$Lc = \frac{{V_{s} }}{{A_{s} }}$$

where the characteristic length of the solid is expressed by $Lc$[*m*]; the volume of the solid is expressed by $V_{s}$[m^3^], and the surface area of heat exchange is expressed by $A_{s}$[m^2^].

Thermal diffusivity:

2 $$\kappa = \frac{k}{{\rho C_{v} }}$$

where $\kappa$ is the thermal diffusivity in [m^2^/s]*; k* is the thermal conductivity in [W/(m·°C)]*; ρ* is the density in [kg/m^3^]; and *C*_*v*_ is the specific heat at constant volume in [J/kg·°C].

Characteristic time:

3 $$t_{c} = \frac{{L_{C}^{2} }}{\kappa }$$

where the characteristic time of the solid is expressed by $t_{c}$[*s*].

The differential expression of the energy balance has the following form:

4 $$- q_{i,i} + q_{v} = \frac{\partial \zeta }{{\partial t}}$$

where *q*_*i,i*_ is the heat-flux vector in [W/m^3^]; *q*_*v*_ is the volumetric heat-source intensity in [W/m^3^], and *ζ* is the heat stored per unit volume in [J/m^3^].

In general, the temperature change may be caused by variations in both energy storage and volumetric strain *ε*. The constitutive thermal law relating those parameters may be expressed.

as:

5 $$\frac{\partial T}{{\partial t}} = M_{th} (\frac{\partial \zeta }{{\partial t}}{ - }\beta_{th} \frac{\partial \varepsilon }{{\partial t}})$$

where *M*_*th*_ and *β*_*th*_ are material constants and *T* represents the temperature.

In this law, a particular case of *β*_*th*_ = 0 and *M*_*th*_ = $\frac{{1}}{{\rho C_{v} }}$ is considered, in which *ρ* is the mass density of the medium in [kg/m^3^] and *C*_*v*_ is the specific heat at constant volume in [J/kg·°C]. The change in strain is assumed to play a minor role in influencing the temperature validity for quasistatic mechanical problems involving solids and liquids.

6 $$\frac{\partial \zeta }{{\partial t}} = \rho C_{v} \frac{\partial T}{{\partial t}}$$

By substituting Eq. (6) for Eq. (4), the energy-balance equation was yielded.

7 $$- q_{i,i} + q_{v} = \rho C_{v} \frac{\partial T}{{\partial t}}$$

For all solids and liquids, the specific heats at constant pressure and constant volume are principally equivalent. Accordingly, *C*_*v*_ and *C*_*p*_ can be used by each other.

According to the finite-difference approximation principle of spatial derivatives, the numbers from 1 to 4 represent each node of the tetrahedron, the opposite side of node n is face n, and the value of the superscript (f) is related to the relevant variable on the face f.

The temperature changes linearly in the tetrahedron. The temperature gradient is represented by the node value of temperature according to the Gauss divergence theorem:

8 $$T,_{j} = - \frac{1}{3V}\mathop \sum \limits_{l = 1}^{4} T^{l} n_{j}^{(l)} S^{(l)}$$

where the external unit vector perpendicular to surface *l* is denoted by [*n*]^*(l)*^, the surface area is denoted by *S*, and the tetrahedral volume is denoted by *V*.

Energy-balance equation formula of nodes. The energy-balance Eq. (7) may be expressed as:

9 $$q_{i,i} + b* = 0$$

where

10 $$b* = \rho C_{v} \frac{\partial T}{{\partial t}} - q_{v}$$

is the instantaneous "physical strength" in the mechanical node formula. Using a tetrahedron analogy, the nodal heat $Q_{e}^{n} [w]$ n = 1,4 in equilibrium with its heat flux and body force can be expressed as:

11 $$Q_{e}^{n} = Q_{t}^{n} { - }\frac{{q_{v} V}}{{4}} + m^{n} C_{v}^{n} \frac{{{\text{d}}T^{n} }}{dt}$$

where

12 $$Q_{t}^{n} = \frac{{q_{i} n_{i}^{(n)} S^{(n)} }}{3}$$

and

13 $$m^{n} = \frac{\rho V}{{4}}$$

In this theory, the node form of the energy-balance equation is required at each global node, in which the sum of equivalent node heat ($- Q_{e}^{n}$) of all tetrahedrons and the node contribution ($- Q_{w}^{n}$) of the applied boundary flux and source are zero.

In heat convection, it is presumed that fluid flow occurs within saturated fractures while the rock matrix is impermeable. As described in the previous section, heat can be transported by fluid convection, conducting in itself, and the rock mass. The fluid temperature generally varies in different rocks. Therefore, between the fracture fluid and the contacting rock (fluid-thermal coupling), heat transfer may occur, according to Newton’s law of cooling. Coupling to heat transfer within the rock and the logic for heat transfer within the fluid is presented as follows.

Heat convection in the flow planes is described by the following equations. Heat is transported.

by conduction in the fracture fluid, according to Fourier’s law:

14 $$q_{f}^{T} = - k_{f}^{T} \Delta T$$

where $q_{f}^{T}$ is the specific heat flux in the fluid in [*W/s*^*2*^] and $k_{f}^{T}$ is the fluid thermal conductivity in [W/(m·°C)]. The energy-balance equation for the fluid obeys the equation.

15 $$\rho_{f} c_{f} \frac{{\partial T_{f} }}{\partial t} + \nabla \cdot q_{f}^{T} + \rho_{f} c_{f} q^{f} \cdot \nabla T_{f} + A_{f} h(T_{f} - T_{s} ) = 0$$

where $\rho_{f} c_{f}$ is the fluid density [kg/m^3^] times the specific *heat [*J/(g·°C*)*]; $q^{f}$ is the specific fluid discharge.

in [m^2^/s]; $A_{f}$ is the contact area per unit fluid volume in [*m*^*2*^]; *h* is the fluid/rock heat transfer coefficient in [W/(m^2^*·*°C)]; and *T*_*f*_ and *T*_*s*_ are the temperatures of the fluid and solid block, respectively.

For the solid blocks, the fluid flow was neglected; the transport of heat obeys Fourier’s law as follows:

16 $$q^{T} = - k^{T} \Delta T$$

where *q*^*T*^ is the specific heat flux in [W/s^2^] and *k*^*T*^ is the rock thermal conductivity in [W/(m·°C)]. The energy balance is

17 $$\rho_{s} c_{s} \frac{{\partial T_{s} }}{\partial t} + \nabla \cdot q_{s}^{T} -  A_{s} h(T_{f} - T_{s} ) = 0$$

where $\rho_{{\text{s}}} c_{s}$ is the solid density [kg/m^3^] times the specific *heat *[J/(g·°C)] and *A*_*s*_ is the contact area per unit volume of solid (from the aspect of fluid, there is contact on two sides: $A_{s}^{ + }$, $A_{s}^{ - }$, and *A*_*s*_ = $A_{s}^{ + }$ + $A_{s}^{ - }$).

## Example model

**Figure 2:**
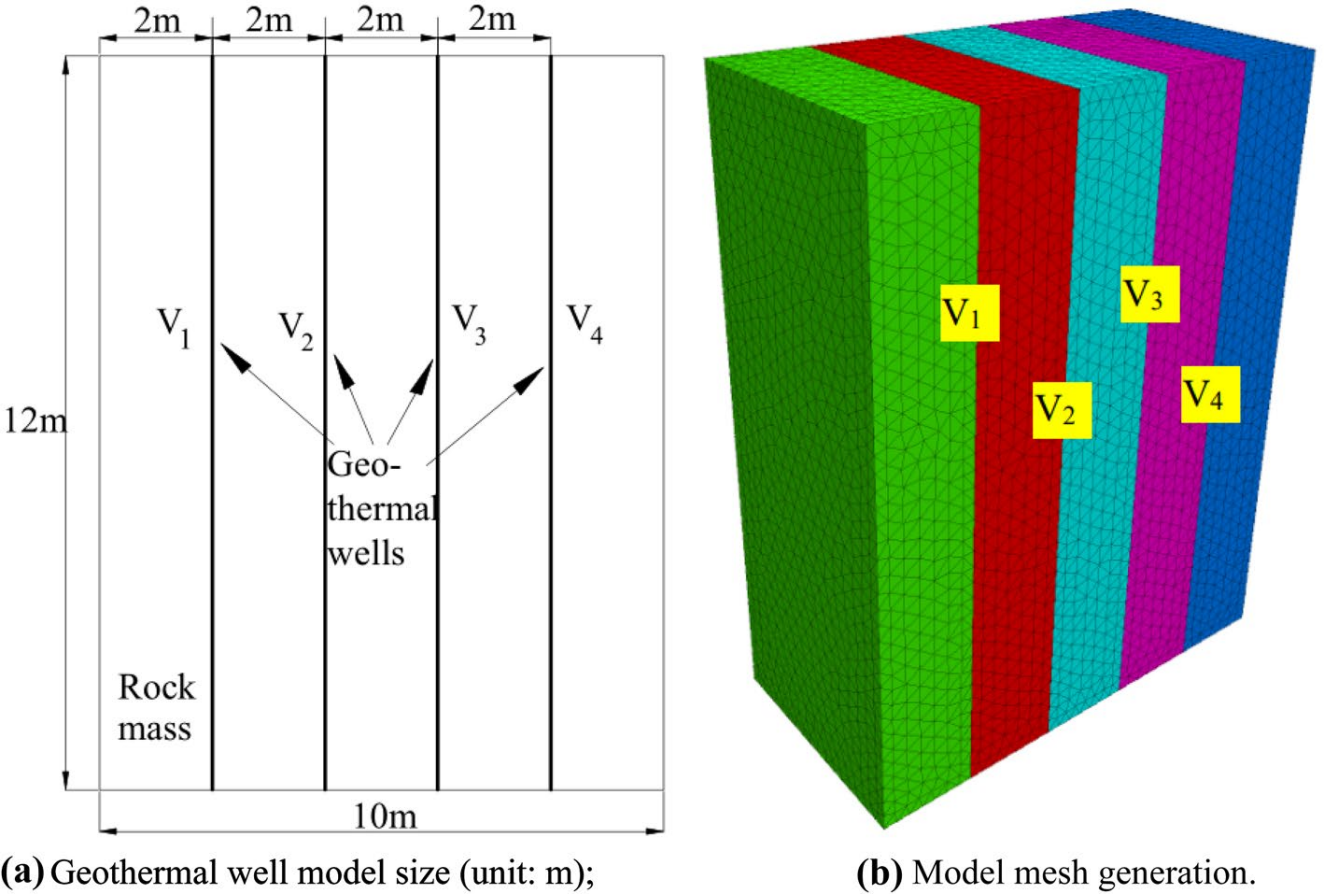
Geothermal well model size and mesh generation.

In this paper, it is assumed that there is a hot rock with well-developed fractures in Northwest China, which has a huge heat reserve but is relatively deficient in groundwater resources. As a result, it proposed to inject water and effluent to ensure the long-term viability of geothermal exploration and provide stable expedition for local businesses. Considering the hydrothermal heat storage at approximately 100 m underground, low-temperature geothermal resources less than 90 °C are used for heating and technological processes. In the process of geothermal exploration, the interaction between the injection well and production well affects the water temperature distribution at the outlet of the production well and the temperature of the rock mass. The geothermal expedition process involves the interaction of injection wells, injection wells and production wells, and production wells on the outlet water temperature of production wells and rock mass temperature. In this paper, it is assumed that there are four geothermal wells in the model, and the optimization mechanism of the thermal recovery efficiency under different geothermal well layout conditions was studied. The model size was 10 m [length] × 5 m [width] × 12 m [height], the spacing between geothermal wells was set at 2 m, and the distance between the geothermal well and model boundary was also set as 2 m. The boundary conditions were as follows: the inlet unit temperature of the production well was set as the geothermal temperature, the outlet unit was set as the free temperature, then the inlet unit temperature of the injection well was set as the normal temperature. The outlet unit was set as the free temperature, and the other sides were adiabatic. The surrounding rock temperature was approximately 20 °C at  − 100 m above the ground, and the model assumed that the initial water temperature of the rock and injection well was 20 °C. The numerical model size and grid division of the optimization study on the thermal recovery efficiency under different geothermal well layout conditions are shown in Fig. 2. Here fractures V_1_, V_2_, V_3_ and V_4_ were simulated in four geothermal wells, with different water injection and water outlets.

## Parameters and content

Under conventional conditions, the thermophysical parameters of rock and water are listed in Table 1, in which the heat convection coefficient of rock and water was 30 W/(m^2^·°C).

**Table 1:** Thermophysical parameters of the rock and water.

Material	Density/(kg/m^3^)	Specific heat/(J/(g·°C))	Coefficient of thermal conductivity/(W/(m·°C))
Rock	2700	0.8	2.3
Water	1000	4.2	0.6

**Figure 3:**
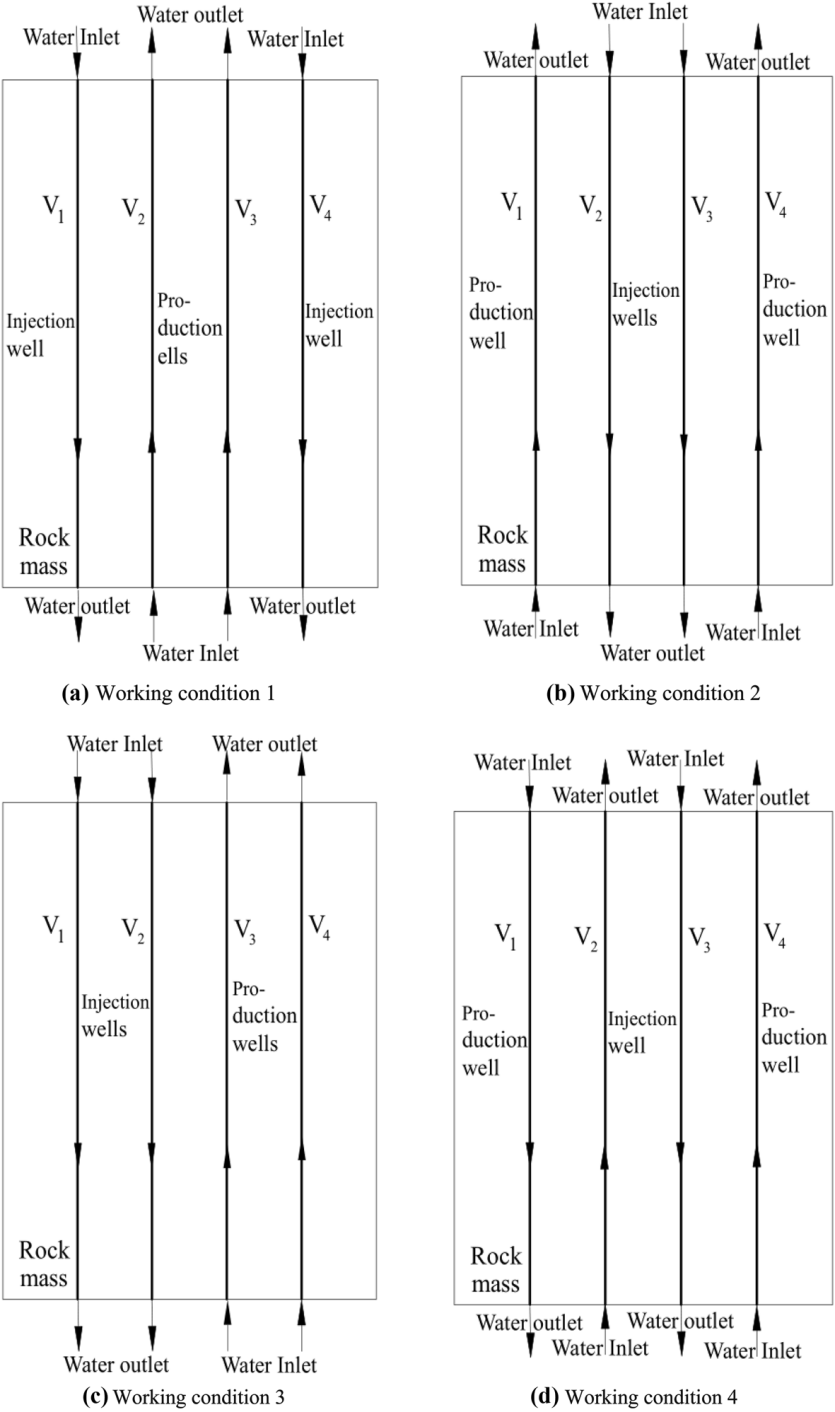
Model calculation conditions.

The calculation conditions of the model are shown in Fig. 3. The calculation was carried out per the principle of establishing the same opening of geothermal wells, ensuring the same flow rate of injection wells and production wells and the same water flow velocity of injection wells and production wells. The calculation contents of the model are shown in Table 2. Here the inlet water temperature of the production well was 90 °C, the fracture opening (production well) was 2.5 mm. Fractures V_1_ and V_4_ were set to inject water, whereas V_2_ and V_3_ were set to outlet water, and the water flow speed was 2 mm/s. The fractures V_1_ and V_4_ were used to outlet water, while V_2_ and V_3_ were used to inject water, and the water flow speed was 2 mm/s. Set fractures V_1_ and V_2_ to inject water and V_3_ and V_4_ to outlet water, with a water flow rate of 2 mm/s. Water was injected into fractures V_1_ and V_3_, and a water flow velocity of 2 mm/s was applied to fractures V_2_ and V_4_. Under these four working conditions, the influence of different water injections and water flows on the heat transfer temperature of the rock mass was simulated, calculated, and analyzed. The data obtained under each working condition were processed by postprocessing software into the rock mass temperature field and water temperature–time curve at the outlet of the production well for comparative analysis.

**Table 2:** Numerical simulation conditions.

Calculation content	Water injection mode	Water velocity/(mm/s)	Inlet water temperature of production well/(°C)	Fracture (geothermal well) opening/(mm)
1	V_1_, V_4_ water injection, V_2_, V_3_ water outlet	2	90	2.5
2	V_1_, V_4_ water outlet, V_2_, V_3_ water injection			
3	V_1_ V_2_ water injection, V_3_, V_4_ water outlet			
4	V_1_, V_3_ water injection, V_2_, V_4_ water outlet			

## Results and discussion

### Influence of different injection wells and outlet wells on the temperature field of the rock mass

**Figure 4:**
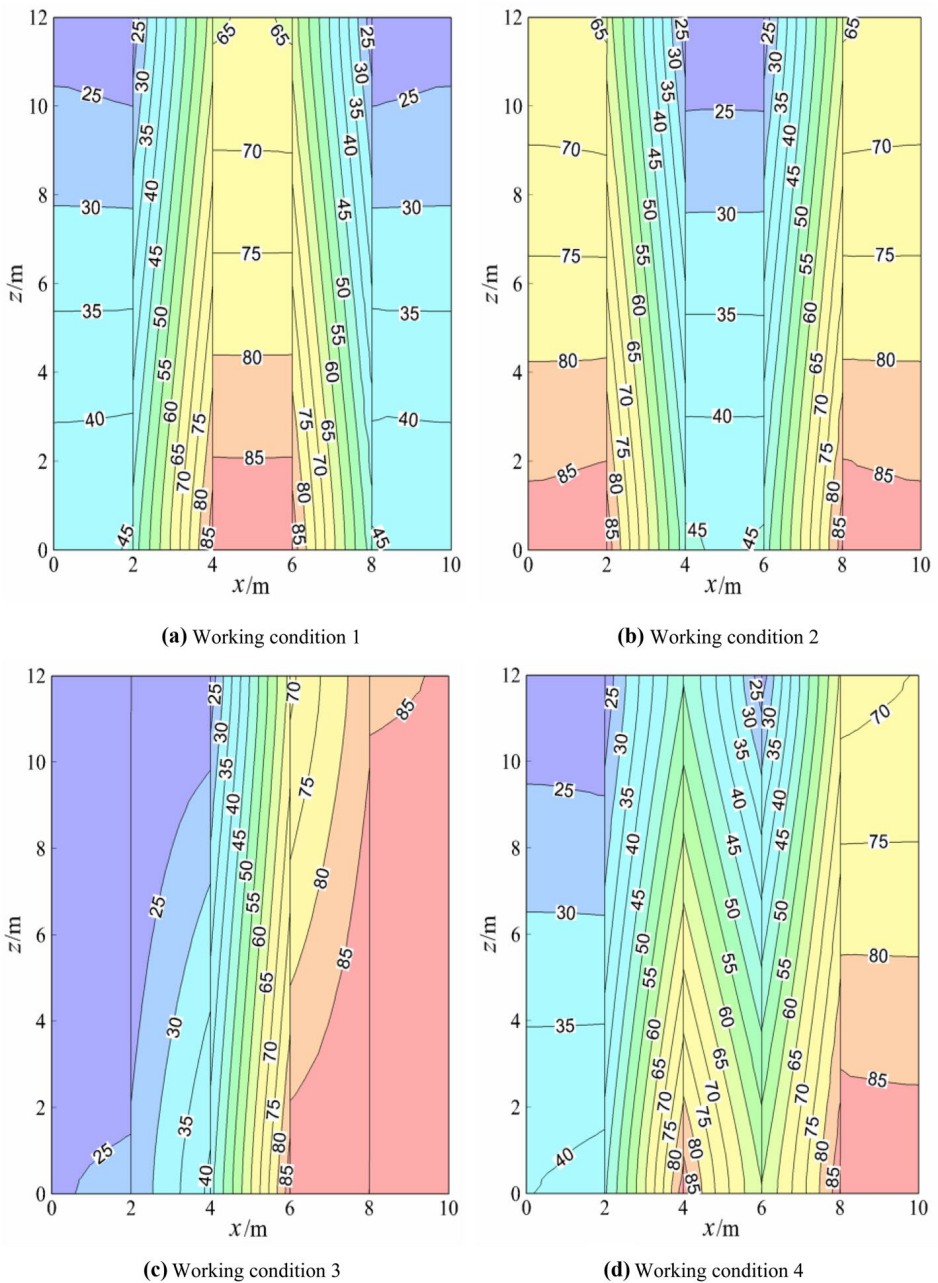
Temperature field of rock mass.

The temperature field of the rock mass is shown in Fig. 4 under four working conditions, when the model reached a steady state.

Figure 4a, b revealed that, in the initial state, low-temperature water (20 °C) was injected into the ground along with injection wells V_1_ (V_2_) and V_4_ (V_3_), while high-temperature water (90 °C) was pumped out from production wells V_2_ (V_1_) and V_3_ (V_4_). When the high-temperature water of production wells V_2_ (V_1_) and V_3_ (V_4_) entered the production well, it convected heat transfer with the rock mass on both sides of the production well (initial 20 °C); that is, the heat absorption temperature of the rock mass on both sides of the production well gradually increased, and the heat release temperature of the water flow of the production well decreased steadily. As the temperature of the rock mass on both sides of the production well increases, the low-temperature water flow of injection wells V_1_ (V_2_) and V_4_ (V_3_) passes through the rock mass with elevated temperature on one side and heat convection occurs between them. The three water flow heat release processes of production wells (heat convection between water flow of production well and its rock mass wall), the production wells water absorbed heat by contacting the rock mass wall (respective heat conduction of production well water and rock mass wall) and water flow heat absorption of injection wells (heat convection between water flow of injection well and its rock mass wall) were accompanied by water injection and water pump until the model reached a uniform state. At this time, the total amount of heat provided by the inlet water of the production wells was equal to the heat absorbed by the rock mass at its sidewall. The heat was absorbed by the water flow of the injection well, and they reached dynamic equilibrium. In addition, the temperature gradient at the edge (middle) of rock under working condition 1 was similar to that at the middle (edge) of rock under working condition 2. After the injection wells and production wells under two working conditions were switched, their temperature gradients were very similar, which constituted axial symmetry. The rock temperature gradients on both sides of the injection well (production well) were about 1.67 °C/m and 4.93 °C/m respectively, and the values of the rock temperature gradients were the same, but the temperature gradients' direction was opposite, which was caused by the same boundary conditions.

Comparison Fig. 4a, c showed that after the middle production well and edge injection well in Fig. 4a were changed to the left adjacent production well and the right adjacent injection well in Fig. 4c, the temperature field of rock mass of both sides of the edge formed a central symmetry, and the temperature gradient from the middle to both sides of the rock mass became smaller and smaller. Also, it showed that the water temperature at the outlet of injection well V_1_ decreased significantly and that at the outlet of production well V_4_ increased significantly, the water temperature at the outlet of injection well V_2_ decreased slightly, while that at the outlet of production well V_3_ increased slightly. This is due to the thermal superposition effect of the adjacent injection well and the production well through the rock mass, which led to the higher temperature of the production well. A comparison between Fig. 4a,d indicated that from the middle production well in Fig. 4a, the edge injection well was changed to the interval between the injection well and production well in Fig. 4d. The temperature field of the rock mass on both sides of the edge formed a central symmetry, and the temperature gradient from the middle to both sides of the rock mass decreased, showing that the water temperature at the outlet of injection well V_1_ decreased slightly and that at the outlet of injection well V_3_ increased significantly. Also, the water temperature at the outlet of production well V_2_ decreased significantly, while the water temperature at the outlet of production well V_4_ changed a little, whereas the temperature gradient mainly showed a large difference between x (4–6 m). This was because in Fig. 4a, the heat superposition effect occurred in the middle production well through the rock mass, forming a temperature gradient from the bottom to the top, while in Fig. 4d, there was no heat superposition effect between the water injection wells and the production wells, but the heat convection between injection wells and the production wells was dominant, and the temperature gradient was mainly formed from left to right in a steady state.

By comparing Fig. 4b with Fig. 4c, after the production well V_1_ and injection well V_3_ in Fig. 4b were changed to injection well V_1_ and production well V_3_ in Fig. 4c, only the temperature field within the range of [x (6–8 m) and z (0–12 m)] in Fig. 4b and [x (4–6 m) and z (0–12 m)] in Fig. 4c was the same. This is due to the "reverse direction" heat superposition of rocks between the injection well and production well under working conditions 2 and 3, resulting in a large temperature gradient of 4.93 °C/m. The temperature gradient of rocks between the injection wells in Fig. 4b was about 1.67 °C/m, and that between injection wells in Fig. 4c was 1.23 °C/m, which indicated that the heat conduction rate of rocks between injection wells in condition 3 was lower than that in condition 2. This is due to the different boundary conditions outside the injection wells. According to the comparison between Fig. 4b,d, after changing from the production well V_1_ and injection well V_2_ in Fig. 4b to injection well V_1_ and production well V_2_ in Fig. 4d, only [x (2–4 m), z (0–12 m)], [x (6–8 m) and z (0–12 m)] in Fig. 4b were the same as those in [x (4–6 m), z (0–12 m)], [x (6–8 m) and z (0–12 m)] in Fig. 4d, which is due to the "opposite direction" heat superposition of rocks between injection wells and production wells in working conditions 2 and 4. In Fig. 4b, the temperature gradients of rocks from the outside to the middle were about 1.67 °C/m、4.93 °C/m and 1.67 °C/m respectively. In Fig. 4d, the temperature gradients of rocks from the outside to the middle were about 1.25 °C/m、4.93 °C/m and 4.93 °C/m respectively. The average temperature gradients under working condition 2 and 4 were about 2.76 °C/m and 3.7 °C/m respectively, indicating that the heat conduction rate of rocks in working condition 4 was higher than that in working condition 2. This is because the rock heat superposition effect between injection wells was less than the rock heat conduction effect between injection wells and production wells. That is, the rock temperature between injection wells was less than that between injection wells and production wells.

Comparing Fig. 4c with Fig. 4d, it can be seen that water injection wells V_2_ and V_3_ in Fig. 4c were changed into production wells V_2_ and V_3_ in Fig. 4d; that is, adjacent injection wells and adjacent production wells were changed into spaced injection wells and production wells. In Fig. 4c, the heat superposition effect between adjacent production wells was dominant, which made the water temperature at the production well outlet greatly increase, while in Fig. 4d, heat convection was dominant in the injection wells and production wells, which greatly decreased the water temperature at the outlet of the production well.

### Temperature field analysis of the water injection well and water outlet well

**Figure 5:**
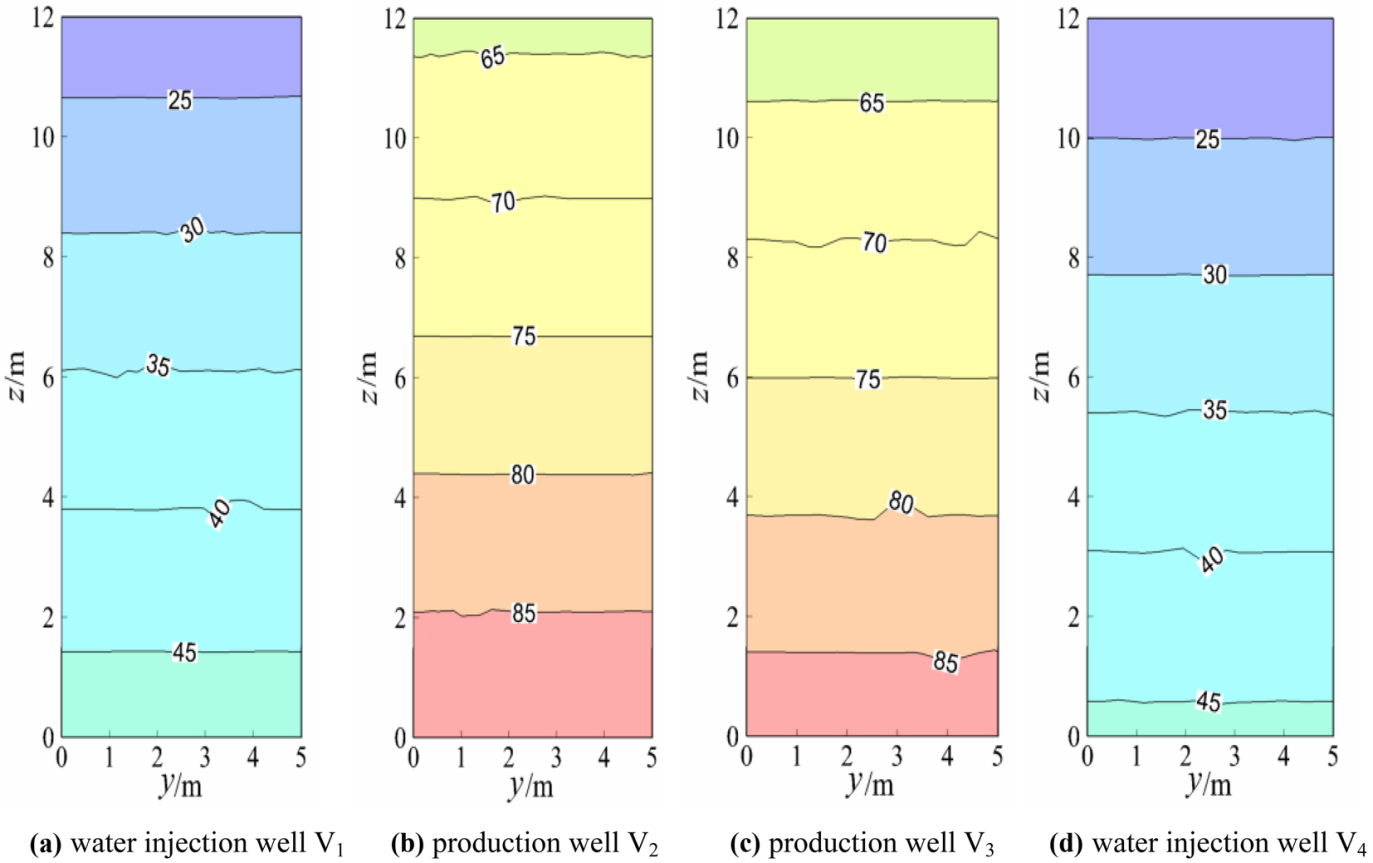
Temperature field of the geothermal well plane (working condition 1).

**Figure 6:**
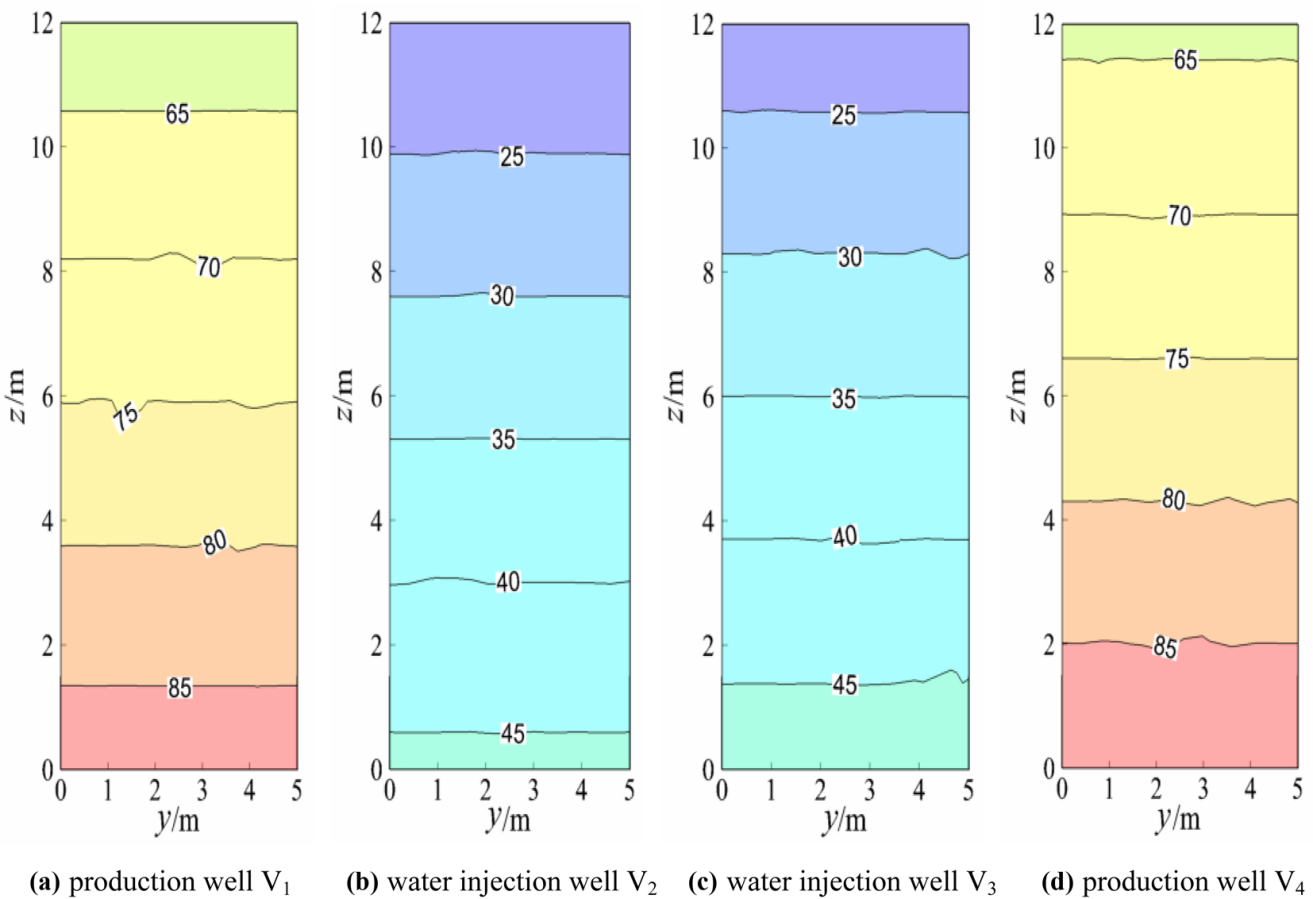
Temperature field ofthegeothermal well plane (working condition 2).

**Figure 7:**
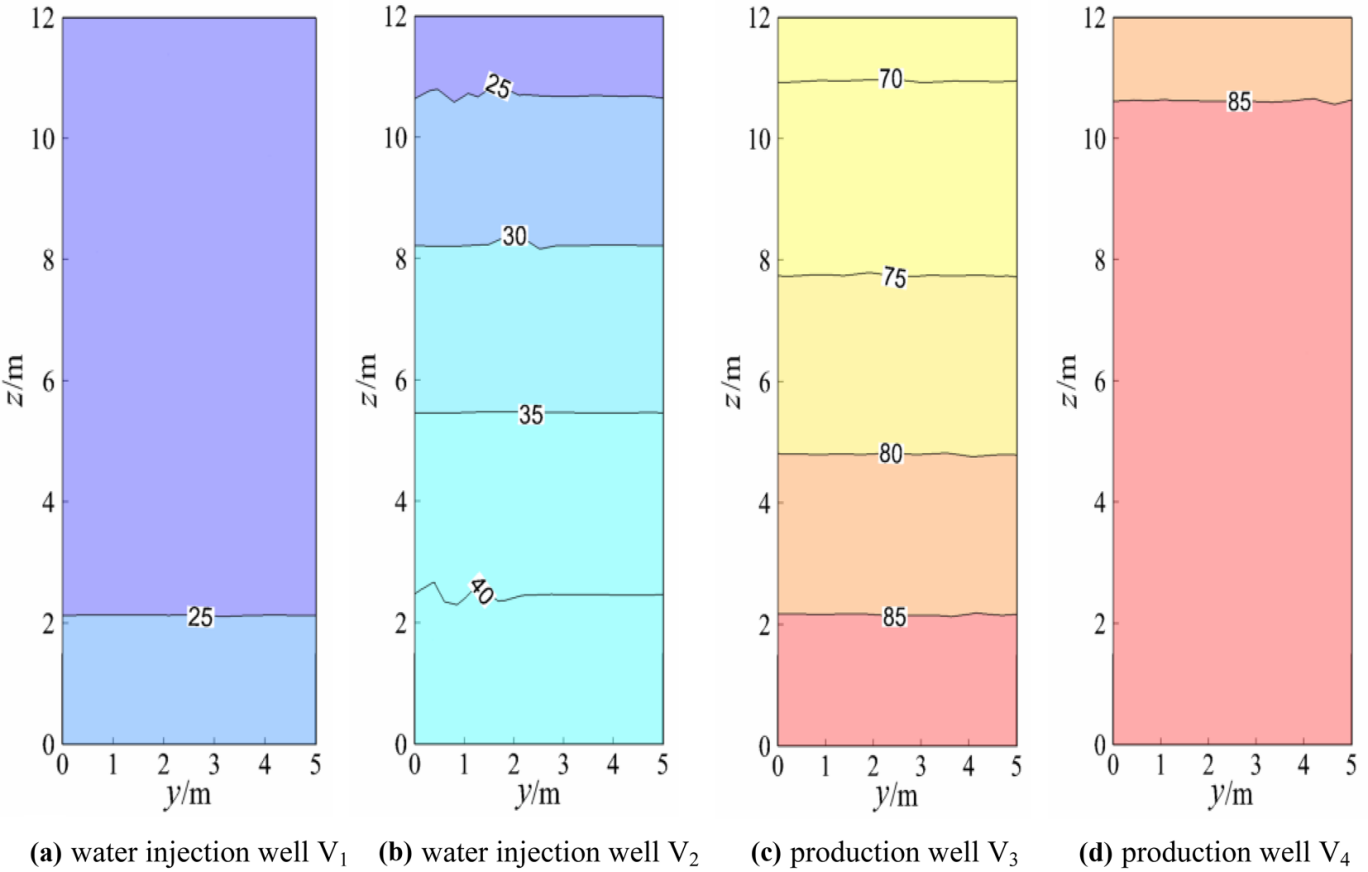
Temperature field of the geothermal well plane (working condition 3).

**Figure 8:**
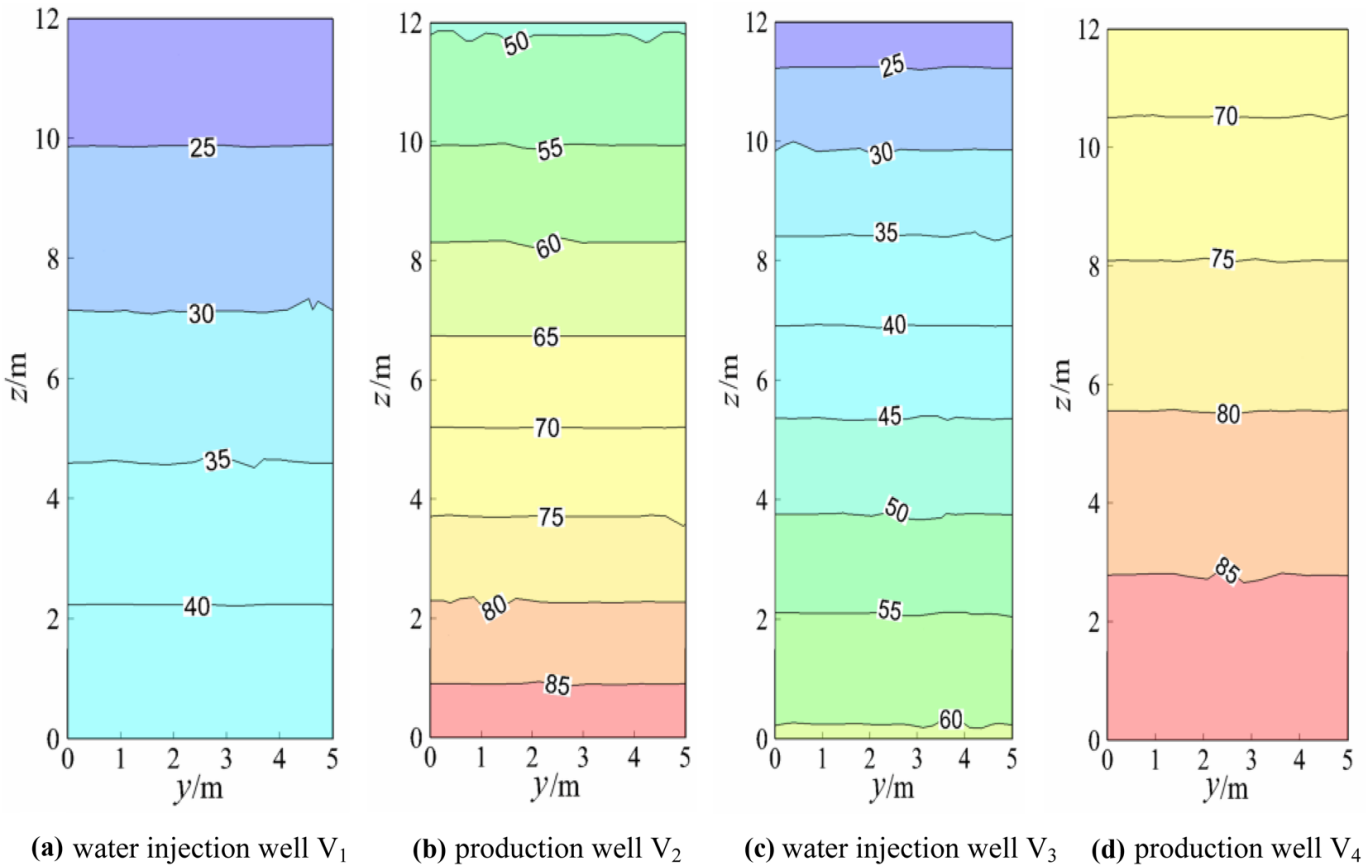
Temperature field of the geothermal well plane (working condition 4).

The temperature fields of the injection wells and water outlet wells are shown in Figs. 5, 6, 7, and 8 when the model reached a steady-state under four working conditions.

When the model reached a steady state, the production wells V_1_ and V_4_ and injection wells V_2_ and V_3_ were symmetrical, and the theoretical isotherms were the same, as shown in Fig. 5. The slight difference between the temperature fields of the production well and injection well was due to the random distribution of the model calculation grid, which had certain errors. The temperature gradients of the overall injection well and the production well were similar, and the temperature gradients of production wells V_1_ and V_4_ and injection wells V_2_ and V_3_ were also almost the same (approximately 2.13 °C/m). Figure 5 and Fig. 6 indicated that after the switch between the production well and water injection well, the temperature gradients of production wells V_1_ and V_4_ and injection wells V_2_ and V_3_ were almost the same (approximately 2.15 °C/m). The temperature gradients of the production well and injection well under the two working conditions were almost the same in numerical terms, but the difference was that the temperature gradients were in opposite directions. Again, Fig. 5 and Fig. 7 showed that after changing from the middle production well and edge injection well in Fig. 5 to the injection well on the left and the production well on the right, the temperature gradients of V_1_ and V_2_ of the injection well were approximately 0.51 °C/m and 1.85 °C/m, respectively. The temperature gradients of production wells V_3_ and V_4_ were approximately 1.69 °C/m and 0.47 °C/m, respectively, and the temperature gradient decreased. The reason for this was that the boundary conditions of injection and production wells had been altered. A comparison between Fig. 5 and Fig. 8 showed that the middle production well and marginal injection well in Fig. 5 were changed into interval injection wells and production wells in Fig. 8, whereas in Fig. 8, the temperature gradients of injection wells V_1_ and V_3_ were approximately 1.95 °C/m and 3.13 °C/m, respectively, and those of production wells V_2_ and V_4_ were approximately 3.18 °C/m and 1.97 °C/m, respectively. The temperature gradients of water injection wells V_1_ and V_4_ and V_3_ and V_2_ were indistinguishable. This is due to the similar boundary conditions between interval injection wells and production wells.

Comparison Figs. 6 and 7 revealed that after the production well V_1_ and injection well V_3_ in Fig. 6 were changed to injection well V_1_ and production well V_3_ in Fig. 7, the temperature field of injection well and production well plane in Fig. 7 changed greatly. The temperature gradient of the injection well and production well in Fig. 6 was 1.67 °C/m whiles the temperature gradient of injection well V_1_ and production well V_4_ in Fig. 7 became 0. The temperature gradient of injection well V_2_ and production well V_3_ was about 1.25 °C/m, indicating that after the two injection wells adjacent to the middle of Fig. 6 became the injection well adjacent to the left and the production well adjacent to the right of Fig. 7, the temperature gradient of the geothermal well decreased, that is, the water flow and heat transfer rate of the geothermal well decreased. When comparing Figs. 6 and 8, the temperature gradient of both injection well and production well in Fig. 6 was 1.67 °C/m, while that of injection well V_1_ and production well V_4_ in Fig. 8 was around 1.25 °C/m. The temperature gradient of production well V_2_ and injection well V_3_ was about 2.92 °C/m, and the average temperature gradient was about 2.09 °C/m, indicating that the temperature gradient of the geothermal well increased after the two injection wells adjacent to the middle part of Fig. 6. The central injection wells adjacent became the interval injection well and production well respectively, implying that the heat transfer rate of water flow increased.

The injection wells V_2_ and V_3_ in Fig. 7 were changed into production wells V_2_ and V_3_ in Fig. 8 based on the comparison of Figs. 7 and 8. That is, adjacent injection wells and adjacent production wells were modified into spaced injection wells and production wells, and the temperature gradient of water flow in injection wells and production wells in Fig. 7 was much smaller than that in Fig. 8. This is because heat convection between spaced injection wells and production wells was dominant and the water flow and the heat transfer speed was faster under the assumption of constant thermal resistance between the rock mass and the contact surface of the water flow.

### Water temperature–time analysis of geothermal well outlet

**Figure 9:**
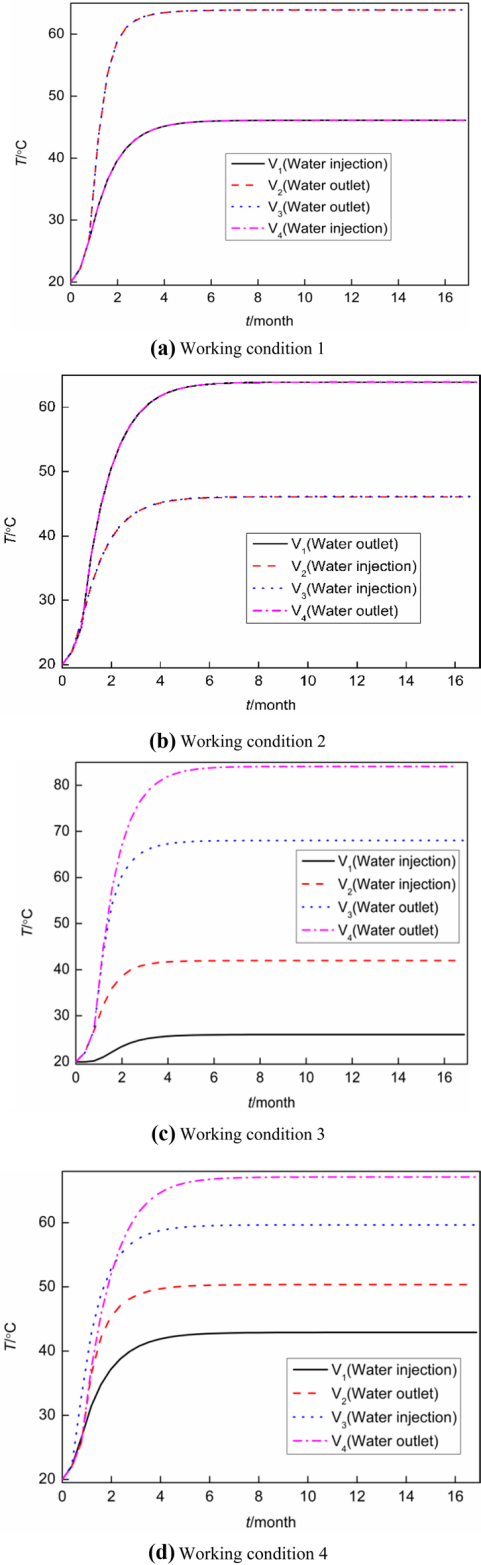
Temperature–time curve of geothermal well outlet.

The temperature–time curve of the geothermal well outlet is shown in Fig. 9 under four conditions.

As shown in Fig. 9a, under the condition that the middle part was production wells and the edge was injection wells, due to the symmetry of the model, the water temperature–time curves of production wells V_1_ and V_4_ and injection wells V_2_ and V_3_ coincided from the beginning to the end. It took approximately 7 months for the model to reach a steady state. At this time, the water temperature at the outlet of the production well reached 63.9 °C, the water temperature at the outlet of the injection well reached 46.1 °C, and the water temperature of the production wells was 38.61% higher than that of injection wells. According to the comparison in Fig. 9a, b, the model under working condition 2 took approximately 7 months to reach a steady-state after switching between injection wells and production wells. At this time, the water temperature at the outlet of production wells V_1_ and V_4_ and injection wells V_2_ and V_3_ was the same as that at production wells V_2_ and V_3_ and injection wells V_1_ and V_4_ in working condition 1. As shown in Fig. 9c, it took approximately 6 months for the model to reach a steady state. At this time, the water temperature at the outlet of production wells V_3_ and V_4_ reached 68.0 °C and 84.0 °C, respectively, and the water temperature at the outlet of injection wells V_1_ and V_2_ reached 41.9 °C and 25.9 °C, respectively. The reason was that the thermal superposition effect of the water flow of the adjacent production well was dominant. The heat absorption capacity of the rock mass boundary on the right side of production well V_4_ (outside of the model was the adiabatic boundary) was less than that on the left side of production well V_3_ (heat absorption capacity of injection well V_2_). Likewise, although some heat superposition effect would occur in the water flow of adjacent production wells V_1_ and V_2_, the heat absorption capacity of the rock mass boundary on the right side of injection well V_2_ (heat release from the production well V_3_) was greater than that on the left side of injection well V_1_ (outside the model was the adiabatic boundary). Also, the water temperature at the outlet of the production well and the injection well changed greatly when production well V_2_ and water injection well V_4_ were changed modified see Fig. 9a–c. This is because the both the left injection wells and the right production wells were adjacent. The water temperature at the outlet of the production well in working condition 3 was 4.1 °C (68.0–63.9 °C) and 20.1 °C (84.0–63.9 °C) higher than that in working condition 1, respectively. The average water temperature at the outlet of the production well in working condition 3 was approximately 12.1 °C (76.0–63.9 °C) higher than that in working condition 1. As shown in Fig. 9d, it took approximately 10 months for the model to reach a steady state. At this time, the water temperature at the outlets of production wells V_4_ and V_2_ reached 67.1 °C and 50.4 °C, respectively, while the water temperature at the outlets of injection wells V_3_ and V_1_ reached 59.7 °C and 42.9 °C, respectively. Therefore, the water temperature of the injection well outlet (59.7 °C) was higher than that of the production wells (50.4 °C) under working condition 4. The reason is after the separation between the injection well and production well, one side of the boundary of the water flow on both sides of the production well V_4_ was the adiabatic boundary of the rock mass, and the other side was the heat absorption boundary of the water flow on injection well V_3_. Both sides of the water flow on the production well V_2_ were injection wells V_3_ and V_1_ (the outer side of production well V_2_ was the endothermic boundary of water flow), so the water temperature at the outlet of production well V_4_ was higher than that of production well V_2_. Both sides of injection well V_3_ were heat release boundaries of production wells V_4_ and V_2_. Therefore, the high-temperature water flow of the two production wells provided the boundary conditions for both sides of injection well V_3_ to absorb more heat. Because the water flow of injection well V_1_ only absorbed heat from production well V_2_ via heat convection, its temperature was the lowest. It can be seen from the comparison of Fig. 9a,d that the water temperature of production wells and injection wells outlet has changed greatly under two working conditions, and water temperature of production wells outlet in working condition 4 was 3.2 °C (67.1–63.9 °C) and  − 13.5 °C(50.4–63.9 °C) higher than that in working condition 1, and the average water temperature at the outlet of production well in working condition 4 was approximately 8.35 °C (67.1 °C -58.75 °C) lower than that in working condition 1, so working conditions 1 and 2 were superior to working condition 4.

Furthermore, a comparison between Fig. 9b, c indicated that after production well V_1_ and injection well V_3_ in Fig. 9b were changed to injection well V_1_ and production well V_3_ in Fig. 9c, the outlet water temperature of production well V_3_ and V_4_ in Fig. 9c was about 4.1 °C (68.0–63.9 °C) and 20.1 °C (84.0–63.9 °C) higher than that of production well V_1_ and V_4_ in Fig. 9b respectively. The water outlet temperature of working condition 3 was about 12.1 °C higher than that of working condition 2 with an average increase of outlet water temperature of production well of about 18.9%. By comparing Fig. 9b with Fig. 9d, after production well V_1_ and injection well V_2_ in Fig. 9b were changed to injection well V_1_ and production well V_2_ in Fig. 9d, the water temperature at the outlet of production well V_2_ and V_4_ in Fig. 9(d) was about − 13.5 °C (50.4–63.9 °C) and 3.2 °C (67.1–63.9 °C) higher than that of production well V_1_ and V_4_ in Fig. 9b, respectively. The average outlet water temperature in working condition 4 was about − 5.15 °C higher than that in working condition 2, and the average increase of outlet water temperature in producing well was about − 8.8%. By comparing Fig. 9c with Fig. 9d, when the water temperature at the outlet of production wells V_2_ and V_4_ in Fig. 9d was about − 17.6 °C (50.4–68.0 °C) and  − 16.9 °C (67.1–84.0 °C) higher than that of production wells V_3_ and V_4_ in Fig. 9c, respectively, after the water temperature at the outlet of production wells V_2_ and V_4_ in Fig. 9c was changed from production well V_2_ and production well V_3_ in Fig. 9c to production well V_2_ and production well V_3_ in Fig. 9d, the average water temperature at the outlet of production well in condition 4 was about − 17.25 °C higher than that in condition 3, and the average increase of water temperature at the outlet of production well was about − 29.36%. According to the comprehensive comparison of Fig. 9a–d, it can be seen that the water temperature at the outlet of the production well, the optimal order of the model was working condition 3 > working condition 1 = working condition 2 > working condition 4. Furthermore, the time required for the model of working condition 3 to reach steady state was the shortest, while the time required for the model of working condition 4 to reach a steady state was the longest.

## Conclusion

In this paper, a new mathematical modeling approach was presented to improve the thermal exploration efficiency under different geothermal well layout conditions. Fractures V_1_ and V_4_ were developed as injection wells whereas V_2_ and V_3_ as production wells. Fractures V_1_ and V_4_ were taken as production wells, V_2_ and V_3_ as injection wells; Fractures V_1_ and V_2_ were constructed as injection wells, V_3_ and V_4_ as production wells; Fractures V_1_ and V_3_ were constructed as injection wells, V_2_ and V_4_ as production wells. Under these four working conditions, the influence of different injection wells and production wells on rock mass temperature was simulated, calculated, and analyzed by the 3DEC program. The calculations revealed that when the position of the model injection well and production well was adjusted, the isothermal number line of rock mass was almost the same in value, but the direction of the water flow and heat transfer was opposite. The maximum water temperature at the outlet of the production well was 84.0 °C due to the thermal superposition effect of the rock mass between the adjacent injection wells and between the adjacent production wells. Conversely, the minimum water temperature at the outlet of the production well was 50.4 °C under working condition 4, which was determined by the convection heat transfer between the water flow and the rock between the interval injection wells and the interval production wells. Under these two working conditions, the isotherms of rock mass on both sides of the edge showed central symmetry, and the temperature gradient gradually decreased from the middle to both ends of the rock mass, indicating that the heat transfer velocity of rock mass gradually decreased from the middle to both ends. Working condition 3 took approximately 6 months to reach a uniform state while working condition 4 took approximately 10. Under working conditions 1 and 2, the water temperature at the outlet of production well and the time required to reach a steady state were between working conditions 3 and 4.
